# New frontiers in nonlinear nanophotonics

**DOI:** 10.1515/nanoph-2024-0396

**Published:** 2024-08-08

**Authors:** Andrey A. Bogdanov, Sergey Makarov, Yuri Kivshar

**Affiliations:** Qingdao Innovation and Development Center, 12428 Harbin Engineering University, Qingdao 266000, Shandong, China; School of Physics and Engineering, ITMO University, St. Petersburg 197101, Russia; Nonlinear Physics Center, Research School of Physics, Australian National University, Canberra, ACT 2601, Australia

Nonlinear optics was born in 1961 when Peter Franken and his colleagues first demonstrated second-harmonic generation from a crystalline quartz plate. This breakthrough was made possible by the invention of the laser, which provided the required power of coherent light. *Nonlinear nanophotonics* started to develop due to the emergence of advanced nanofabrication technologies such as electron-beam lithography, focused ion-beam milling, and advanced deposition methods. These techniques provide fabrication with nanometer precision and control to explore and exploit nonlinear optical effects at the nanoscale. Today, nonlinear nanophotonics is one of the most promising directions of optics having applications in biomedical imaging, environmental sensing, secure communications, quantum computing, and many others.

**Figure 1:**
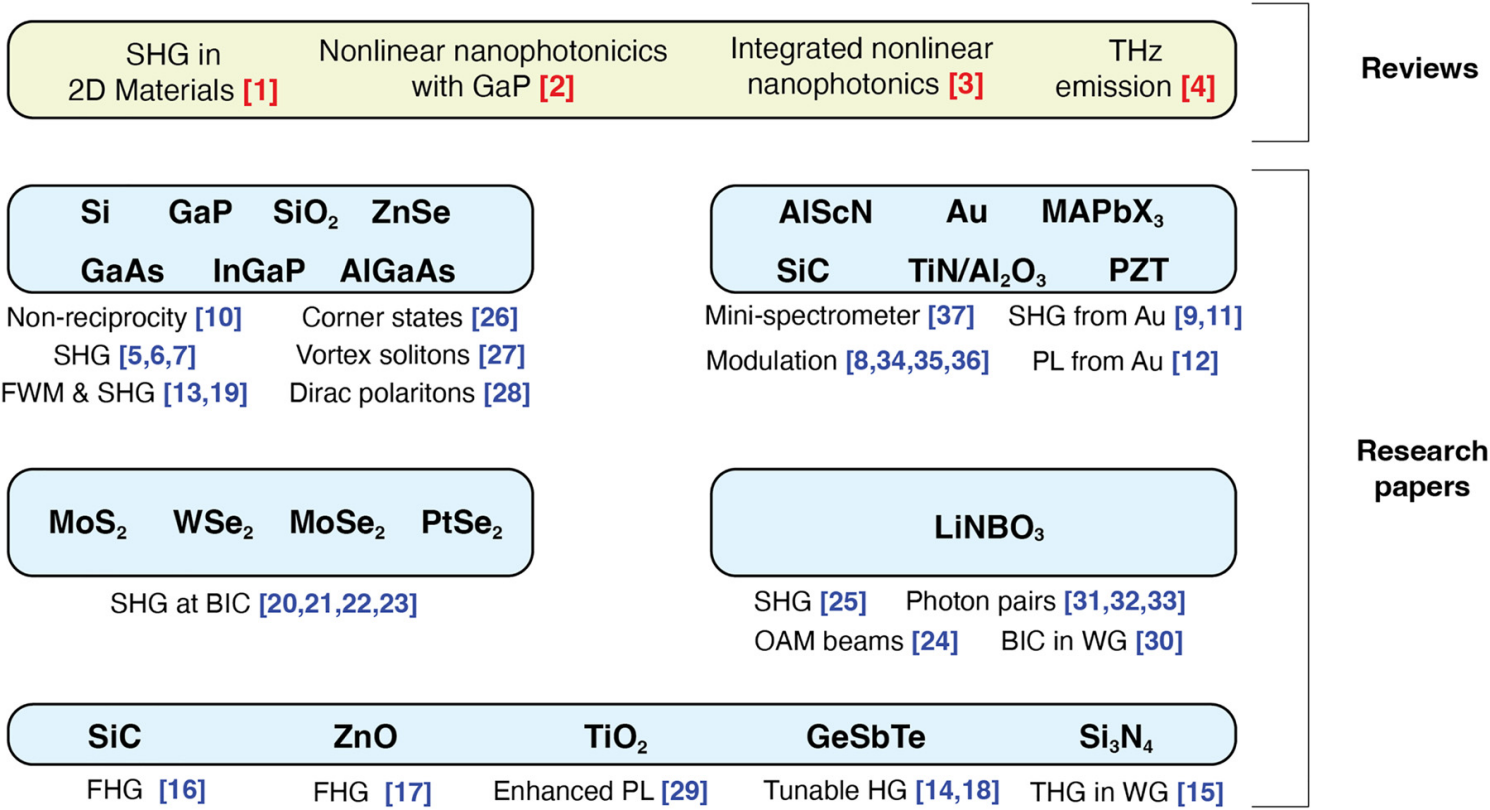
Chart of all papers included in the special issue “New Frontiers in nonlinear nanophotonics”.

This special issue includes an impressive collection of 33 original research papers and 4 topical review papers all devoted to cutting-edge research in nonlinear nanophotonics and the recent progress in this and closely related fields. Figure 1 shows the chart of the papers included to the special issue *New Frontiers in Nonlinear Nanophotonics*.

The review by S. Psilodimitrakopoulos et al. focuses on second harmonic generation (SHG) in 2D materials, particularly metal chalcogenides, and perovskites, discussing polarization-resolved SHG properties and a theoretical framework for nonlinear optical responses [1]. Y. Wang et al. analyze gallium phosphide (GaP) in nanophotonics, highlighting its high refractive index, visible range transparency, and nonlinear optical properties, summarizing material properties, fabrication techniques, and device integration [2]. W. Geng et al. review advancements in nonlinear photonics on integrated platforms, emphasizing CMOS compatibility, energy efficiency, and cost-effectiveness, covering key nonlinear effects and materials, supercontinuum generation, and chip-based optical frequency combs [3]. Y. Lu et al. explore nonlinear optical effects induced by high-power THz sources, highlighting their potential in communications, sensing, and biomedical applications, and reviewing recent developments in THz nonlinear physics and material modulation for novel applications [4].

SHG is one of the most widespread nonlinear effects observed in various nanophotonic structures which is still under active study. In Refs. [5], [6], [7], the authors study theoretically and experimentally the SHG in semiconductors nanostructures including Si nanoparticles, Si/SiO_2_ mesoporous materials, GaP and AlGaAs metasurfaces. An unusual intrinsic nonlinear geometric phase is observed in second harmonic signals from AlGaAs metasurfaces [7]. AlScN is another CMOS-compatible material for integrated nonlinear photonic devices exhibiting enhanced second-order optical nonlinearity that can be used for efficient electro-optical modulation [8]. Plasmonic structures have remained attractive for SHG due to strong localization and large field enhancement. It was shown in Ref. [9] that the second harmonic signal from the plasmonic dimers can be substantially enhanced due to the precise control of the dimer asymmetry. The asymmetry of the scatterer in combination with intensity-dependent permittivity can result in a strong non-reciprocal behavior when the nonlinear scatterer can be cloaked for one excitation direction, yet strongly scatters from the opposite direction [10].

In Ref. [11] (on the front cover of the special issue), the researchers optimize the array pitch for rectangular metagratings composed of V-shaped gold nanoantennas to align diffraction orders with single antenna emissions, thereby maximizing the SHG signal. The photoluminescence (PL) of plasmonic nanoparticles (NPs) has been intensively investigated in recent years and details of this effect are still under discussion. The systematic study of the photoluminescence behavior of plasmonic NPs under varying excitation power levels is presented in Ref. [12]. The authors demonstrate that PL can transition from plasmon-enhanced emission to blackbody-like radiation.

Another second-order nonlinear effect is sum frequency generation (SFG). In Ref. [13], the authors develop a general theoretical framework for multi-objective topology optimization of metasurfaces and report SFG with an efficiency of over 0.2 cm^2^/GW in a 10 nm signal operating bandwidth. A tunable third harmonic generation (THG) is demonstrated using a hybrid dielectric metasurface integrated with the phase-change material Ge_2_Sb_2_Te_5_ (GST) [14]. The phase-matched THG in CMOS-compatible Si_3_N_4_ waveguide is experimentally studied in Ref. [15].

Recent advancements in nonlinear nanophotonics have demonstrated significant progress in controlling and enhancing optical higher-order nonlinear effects at the nanoscale. A SiC metamembrane achieved a two-orders-of-magnitude enhancement in the five harmonic generation (FHG) compared to an unstructured SiC film [16]. Additionally, phase-matching of FWM mixing in ZnO microwires shows a huge enhancement, benefiting from ZnO’s large nonlinear coefficients and wide transparent window indicating the potential for cascaded nonlinear processes [17]. The high-harmonic generation (HHG) in the GST demonstrates reversible optical phase-switching, allowing dynamic control of harmonic emission and introducing GST as a promising material for flexible metasurfaces and ultrafast optical control in integrated photonic devices [18]. In Ref. [19], the authors demonstrate independent geometric control of multiple quasi-bound states in the continuum and their interaction through wave mixing processes, opening new research pathways in nanophotonics with potential applications in information multiplexing, multi-wavelength sensing, and nonlinear imaging.

2D materials exhibit strong light–matter interaction despite their atomic thickness. This results in strong absorption, emission, and nonlinear optical properties, making them suitable for various optoelectronic applications such as photodetectors, light-emitting devices, modulators, and nonlinear photonic components. In Refs. [20], [21], [22], the authors study the enhancement of nonlinear optical properties of MoSe_2_, MoS_2_, WSe_2_ due to interaction with metasurfaces supporting high-Q resonances. In Ref. [23], it was shown that 4-layer PtSe_2_ demonstrates a giant nonlinear response surpassing the second harmonic signal from mechanically exfoliated MoS_2_ by approximately two orders of magnitude. Another prospective material for nonlinear nanophotonics is LiNbO_3_. In Ref. [24] the authors demonstrate the nonlinear generation of optical angular momentum beams with high efficiency across octave-separating wavelengths using the LiNbO_3_ nonlinear photonic crystal platform. In Ref. [25], the authors overcome some challenges in fabricating high-quality ferroelectric domains in thin film lithium niobate ridge waveguides using optimization of the applied electric field distribution. This allows them to achieve the normalized conversion efficiency of about 1,720 % W^−1^ cm^−2^, which is close to 60 % of the theoretical limit. In Ref. [26], the authors demonstrate enhancement of SHG and THG from the Kekule topological corner states in honeycomb dielectric metasurface, which is also not influenced by the geometry shape of the corner. The existence of thresholdless vortex solitons trapped at the core of disclination lattices, which act as higher-order topological insulators, is demonstrated in Ref. [27]. These solitons exhibit strong field confinement and enhanced stability due to their topological nature, with their propagation constant and localization controllable by power.

Bound states in the continuum (BICs) possess exceptionally high Q-factors and strong spatial confinement, which significantly amplify light–matter interactions and nonlinear optical responses at the nanoscale. In Ref. [28], photonic crystal gratings utilize BICs to achieve strong coupling and ultralow threshold condensation of exciton–polariton with unique Dirac-like dispersion. Dielectric BIC metasurfaces have been employed to enhance the radiative properties of coupled J-aggregate films, achieving a 5-fold Purcell enhancement in luminescence intensity and narrowed emission directivity, showcasing the potential for cooperative phenomena in excitonic systems [29]. Novel CMOS-compatible structures comprising two low-refractive-index waveguides on a higher-RI slab (lithium niobate or silicon nitride) support BICs, which offer high-quality factors without stringent geometric control, enabling ultranarrow-bandwidth filters for nanophotonic circuits or a platform for nonlinear nanophotonic components [30].

Photonics offers significant advancements in quantum information processing (QIP), including room-temperature operation, scalability of nanophotonics, and ultrafast operation due to access to ultrabroad bandwidths. Demonstrating a femtosecond biphoton source in dispersion-engineered periodically poled lithium niobate nanophotonics, researchers achieved 17 THz bandwidth with high brightness, paving the way for scalable ultrafast QIP [31]. A new formalism for spontaneous parametric down-conversion in thin films was developed, enabling detailed studies of entangled photon-pair generation and the effects of Fabry–Pérot interferences [32]. Additionally, nonlinear metasurfaces were demonstrated as a platform for precise control of photon pair emission angles, showcasing angularly tunable pair generation with a high coincidence-to-accidental ratio [33].

The development of all-optical modulators is critical for advancing future information processing technologies, as they eliminate the need for electro-optical converters that limit modulation bandwidth. The proposed on-chip ultrafast all-optical modulator utilizing a photonic topological insulator and metallic quantum allows overcoming many limitations related to modulation efficiency, bandwidth, and compact size [34]. It is shown in Ref. [35] that the Raman scattering in crystalline Si nanoparticles is nonlinearly dependent on local temperature due to strong photo-thermo-optical interactions that can be used for efficient all-optical modulation at the nanoscale. Despite the high perspectives of all-optical modulators, the electro-optical ones are still under active study. The electro-optical modulator explored thin-film lead zirconate titanate substrates for on-chip plasmonic electro-optic modulators, achieving over 40 % modulation depth [36]. The integration of perovskite materials with CMOS sensor chips in a miniature spectrometer demonstrates advanced material engineering at the nanoscale that is also prospective for nanophotonic applications [37].

In summary, we believe this special issue *New Frontiers in Nonlinear Nanophotonics* provides valuable snapshots of the current activities of the leading research groups in the field of nonlinear nanophotonics, collecting original articles and review papers on nonlinear optical effects in various nanophotonics structures, and also the discussions of their applications. We hope this collection could stimulate further development of nonlinear nanophotonics and attract more scientists to this rapidly developing research area.

We warmly thank all the authors of this special issue for accepting our invitation and for their valuable contributions, and also extend special thanks to Tara Dorrian and Dennis Couwenberg for suggesting the idea of this project and strongly encouraging and supporting our work during the editorial process.
